# Progress in Research on Alleviating the Symptoms Associated With Advanced Cancer Using Traditional Chinese Medicine

**DOI:** 10.1155/prm/7197339

**Published:** 2026-01-12

**Authors:** Chunmeng Jiao, Ting Zhang, Yachen Yang, Ruofan Zhang, Wenbo Liu, Yanqing Wang

**Affiliations:** ^1^ Chinese Medicine Research Institute, Guangdong Pharmaceutical University, Guangzhou, 510006, China, gdpu.edu.cn; ^2^ Department of Integrative Medicine, Huashan Hospital Affiliated to Fudan University, Shanghai, 200040, China, huashan.org.cn; ^3^ Department of Integrative Medicine and Neurobiology, School of Basic Medical Sciences, Institutes of Integrative Medicine, Fudan University, Shanghai, 200032, China, fudan.edu.cn; ^4^ Shanghai Key Laboratory for Acupuncture Mechanism and Acupoint Function, Shanghai Research Center for Acupuncture and Meridian, Fudan University, Shanghai, 200433, China, fudan.edu.cn

**Keywords:** advanced cancer treatment, symptoms of advanced cancer, Traditional Chinese Medicine

## Abstract

Advanced cancer continues to pose a substantial global challenge, with complex symptom burdens and limited therapeutic options. Traditional Chinese Medicine (TCM), grounded in holistic theory and the principles of syndrome differentiation, employs interventions such as herbal medicine, acupuncture, moxibustion, and acupoint‐based therapies to address both the malignancy and the patient’s overall functional status. Emerging evidence indicates that TCM may alleviate symptom clusters associated with advanced cancer, enhance quality of life, and potentially contribute to improved survival outcomes. This review synthesizes findings from the past decade on the role of TCM in advanced cancer care, with a focus on herbal decoctions, Chinese herbal injections, acupuncture—either alone or in combination with herbal therapy—moxibustion with adjuvant medication, and other external TCM modalities. Evidence is examined regarding their effects on cancer‐related pain, fatigue, gastrointestinal dysfunction, chemotherapy‐ and radiotherapy‐induced toxicities, and immune modulation. By consolidating current clinical and mechanistic insights, this review aims to inform future research and support the integration of evidence‐based TCM approaches into contemporary oncology practice.

## 1. Introduction

Cancer is one of the leading causes of mortality worldwide [1–3]. According to the National Cancer Institute, advanced cancer denotes a clinical stage characterized by accelerated tumor growth and dissemination to lymph nodes or distant organs. Patients with advanced cancer often experience symptoms such as pain, fatigue, weight loss, decreased appetite, nausea, sleep disturbances, anxiety, and depression [4, 5]. Current standard treatments for cancer include surgery, radiotherapy, chemotherapy, targeted therapy, immunotherapy, and combination therapies [6–9]. However, these conventional approaches have significant limitations. Surgery, while often curative, is invasive and carries substantial perioperative risks [10]; incomplete resection may leave minimal residual disease (MRD), increasing the risk of recurrence and metastasis. Radiotherapy and chemotherapy remain mainstays of treatment, inhibiting tumor cell proliferation and invasion, but their nonselective cytotoxicity damages normal tissues, causing toxicities such as myelosuppression, mucosal injury, and gastrointestinal (GI) dysfunction [11]. While immunotherapy and targeted therapy offer greater precision [12], they also cause severe adverse effects and drug resistance and impose substantial economic burdens [13–15]. Critically, treatment‐related adverse events—including pain, fatigue, anorexia, weight loss, nausea, insomnia, anxiety, and depression—often overlap with symptoms of advanced cancer itself [16]. This exacerbates the overall symptom burden, further deteriorating quality of life and compromising treatment adherence [17].

In light of these constraints, there has been a burgeoning global interest in complementary and integrative medical strategies aimed at augmenting therapeutic efficacy while attenuating treatment‐related toxicities. Among them, Traditional Chinese Medicine (TCM), a typical representative of complementary and alternative medicine, has attracted increasing attention as an adjunctive approach in cancer care. Grounded in a holistic philosophy and characterized by multitarget therapeutic actions, TCM has been documented to alleviate a broad spectrum of symptoms associated with advanced cancer, such as pain, fatigue, anorexia, GI dysfunction, and sleep disturbances. Concurrently, it mitigates the adverse effects induced by chemotherapy and radiotherapy, thereby enhancing patients’ overall quality of life [18, 19].

Grounded in classical medical scriptures, exemplified by the *Yellow Emperor’s Inner Canon* (*Huangdi Neijing*), TCM integrates fundamental principles, including “fortifying the body’s defensive Qi to expel pathogenic factors (FuZhengQuXie),” “addressing both the root and the branch symptoms,” “treating the same disease with different methods,” and “treating different diseases with the same methods” into the realm of cancer prevention and therapeutic interventions [20]. From this perspective, cancer etiopathogenesis is ascribed to two core mechanisms: “vital Qi deficiency,” reflecting compromised innate resistance and regulatory functions, and “toxin accumulation with stagnation,” representing obstructed Qi and blood circulation that disrupt physiological balance [21]. Accumulating evidence suggests TCM modalities—herbal medicine, acupuncture, tuina (therapeutic massage), and topical herbal applications—may improve clinical outcomes in advanced cancer, mitigate treatment‐related adverse effects, and enhance quality of life [20, 22, 23]. Investigating TCM’s role in managing advanced cancer symptoms thus holds significant clinical value. Furthermore, molecular studies of key TCM syndromes (Zheng), such as Xue‐Yu (blood stasis), Shi‐Re (dampness‐heat), Yin‐Xu (Yin deficiency), and Pi‐Xu (spleen deficiency), have illuminated biological mechanisms underlying cancer pathogenesis and symptom manifestation [20, 22, 23]. This review synthesizes clinical evidence and mechanistic insights into TCM for advanced cancer‐related symptoms, identifies convergent biological pathways, and explores integration with modern oncologic therapies.

## 2. Search Strategy

A comprehensive literature search was conducted across both English and Chinese academic databases, including PubMed, Google Scholar, CNKI (China National Knowledge Infrastructure), and Wanfang Data, using search terms such as “Traditional Chinese Medicine,” “advanced cancer treatment,” and “symptoms of advanced cancer.” Additional relevant studies were identified through manual screening of reference lists from selected publications.

Given the focus on TCM for advanced cancer symptoms, we considered English and Chinese language articles investigating TCM interventions for symptom alleviation or for reducing adverse effects of conventional therapies. Studies meeting these thematic criteria were included for this synthesis.

## 3. TCM for the Management of Symptoms and Therapy‐Associated Adverse Effects in Advanced Cancer

This section reviews TCM management of advanced cancer symptoms and adverse effects of conventional therapies (e.g., chemotherapy and radiotherapy), discussing internal herbal medicine and external therapies for each major symptom cluster.

### 3.1. Cancer Pain

Cancer pain frequently manifests in advanced‐stage malignancy [24]. From a TCM perspective, its pathogenesis involves the accumulation of cancer toxins, phlegm‐stasis entanglement, Qi and blood stagnation, and meridian obstructions [25]. TCM alleviates cancer pain and improves patients’ quality of life through mechanisms that directly correspond to specific molecular and physiological pathways. The principle of “promoting Qi movement and invigorating blood” is believed to modulate inflammatory mediators, inhibit cyclooxygenase‐2 (COX‐2) and prostaglandin E2 (PGE2) production, and attenuate neuropathic pain by downregulating transient receptor potential vanilloid 1 (TRPV1) and ankyrin 1 (TRPA1) channels in sensory neurons [26–29]. Likewise, clearing heat and detoxification suppresses nuclear factor‐kappa B (NF‐κB) signaling and reactive oxygen species (ROS) generation, thereby mitigating oxidative stress and neuroinflammation [30, 31]. Collectively, these actions interrupt pain signal transduction, diminish peripheral and central sensitization, and restore neuro‐immune homeostasis. These therapeutic approach reflects core TCM principles: syndrome differentiation, holistic regulation, and eliminating pathogenic factors to restore internal balance [25].

#### 3.1.1. Herbal Medicine and TCM Formulas for the Management of Cancer Pain

TCM employs targeted herbal strategies for managing cancer pain based on different syndrome patterns. For Qi stagnation type pain, Qi‐regulating and blood‐activating herbs such as *Corydalis yanhusuo* (*Corydalis yanhusuo* W. T. Wang) and *Cyperus rotundus* (*Cyperus rotundus* L.) are commonly selected to promote Qi flow, resolve stasis, reduce swelling, and relieve pain. In blood stasis‐type cancer pain, blood‐activating and heat‐clearing detoxifying herbs such as *Salvia miltiorrhiza* (*Salvia miltiorrhiza* Bunge), *Sparganium stoloniferum* (*Sparganium stoloniferum* Buch.‐Ham.), and *Corydalis saxicola* (*Corydalis saxicola* Bunting) are used. Cold obstruction and Yang deficiency pattern are treated with warming and blood‐activating herbs such as *Aconitum carmichaelii* (*Aconitum carmichaelii* Debeaux) and *Curcuma longa* (*Curcuma longa* L.) [32, 33]. For phlegm‐damp accumulation, warming, phlegm‐resolving, and diuretic herbs that fortify the spleen and transform dampness are utilized, which include Gastrodia elata Blume (Rhizoma Gastrodiae) and *Aconitum carmichaelii* Debeaux (*Radix Aconiti Lateralis Preparata*) [34]. For heat toxin‐type pain, heat‐clearing and detoxifying herbs such as Dandelion (*Taraxacum mongolicum* Hand.‐Mazz.), Honeysuckle Flower (*Lonicera japonica* Thunb.), and Glabrous Greenbrier Rhizome (*Smilax glabra* Roxb.) are used. Additionally, tonic herbs are often incorporated to supplement Qi and blood, regulate ying‐wei dynamics, and nourish yin and fluids, including Polygonum multiflorum (*Polygonum multiflorum* Thunb.), Codonopsis root (*Codonopsis pilosula* (Franch.) Nannf.), Astragalus root (*Astragalus membranaceus* (Fisch.) Bge.), prepared Rehmannia root (*Rehmannia glutinosa* (Gaertn.) Libosch.), Glehnia root (*Glehnia littoralis* F. Schmidt ex Miq.), and Rehmannia root (Raw) (*Rehmannia glutinosa* (Gaertn.) Libosch.) [35–37]. Recent studies have identified multiple bioactive constituents from these herbs with demonstrated potential for cancer pain relief. Key components include dehydrocorydaline (DHC) from Corydalis yanhusuo, α‐cyperone from Cyperus rotundus, tanshinone IIA (TSN IIA) from Salvia miltiorrhiza, Corydalis Saxicola Bunting total alkaloids from Corydalis saxicola, lappaconitine from Aconitum carmichaelii, curcumin from Curcuma longa, and tetrahydroxystilbene glucoside from Polygonum multiflorum [35, 36, 38–50]. These compounds operate through distinct mechanisms: DHC suppresses microglial activation [51], while TSN IIA has been shown to attenuate cancer‐induced bone pain in a dose‐dependent manner by suppressing spinal HMGB1‐mediated neuroinflammation—reflected by reduced IL‐1β, TNF‐α, and IL‐6 expression—and by inhibiting the hyperexcitability of wide dynamic range neurons in the deep dorsal horn, thereby diminishing both ongoing and breakthrough pain [52]. Curcumin modulates the endocannabinoid system and downregulates TRPV1 to attenuate thermal hyperalgesia [53, 54]. These actions translate the TCM principles of “invigorating blood” and “clearing heat” into measurable neuro‐immune interactions. However, these herbal medicines may be associated with hepatotoxicity, nephrotoxicity, reproductive toxicity, and cardiotoxicity [35, 49, 55–57], with some also demonstrating potential phototoxicity and genotoxicity [58–60]. Therefore, systematic evaluation and rigorous monitoring of the safety of these herbal medicines are still necessary.

In clinical trials, Chinese herbal decoctions have demonstrated efficacy in the management of cancer pain (Table 1). Yiqi Huoxue decoction (Qi‐invigorating and blood‐activating decoction) can alleviate pain caused by cancer‐related fatigue (CRF) [61]. Other herbal formulas such as Xuefu Zhuyu decoction (blood mansion invigorating decoction) from the blood‐activating and stasis‐dispelling category [62], Chaihu Shugan powder and Jiawei Xiangsha Liujunzi decoction (modified six‐gentlemen decoction) from the Qi‐regulating category [63, 64], Shenling Baizhu powder (Ginseng root (*Panax ginseng* C. A. Meyer) and Poria cocos (Poria cocos (Schw.) Wolf) from the Qi‐tonifying category [65], and the empirical formula Gutongling Formula (bone pain relief formula) all can be used for the treatment of cancer pain [66, 67].

**Table 1:** Clinical applications of traditional Chinese medicine formulas in cancer pain management.

Name of the formula	Composition	Indications/syndrome type	Study design	Sample size	Efficacy evaluation
Yiqi Huoxue decoction [61]	Astragalus root, Coix seed, Angelica root, Chuanxiong rhizome, red Peony root, Spatholobus stem, Curcuma longa, Curcuma root, Pinellia rhizome, Inula flower, *Oldenlandia diffusa*, and Actinidia roots	Cancer pain due to Qi and blood deficiency	RCT	82 patients	Yiqi Huoxue decoction significantly alleviated pain symptoms and cancer‐related fatigue in patients with tumors.
Xuefu Zhuyu decoction [62]	Bupleurum root, Angelica root, Rehmannia root, red Peony root, safflower, peach kernel, bitter orange, licorice root, Chuanxiong rhizome, Achyranthes root, and platycodon root	Cancer pain due to blood stasis obstructing the collaterals	P‐RCT	240 patients	Xuefu Zhuyu decoction combined with standardized analgesic treatment alleviated pain in patients with lung cancer and significantly reduced their intake of opioid analgesics.
Modified Xiangsha Liujunzi decoction [64]	Costus root (added later), amomum fruit (added later), Atractylodes rhizome, tangerine peel, Pinellia rhizome, Codonopsis root, Cassia seed, Poria, licorice root, and Astragalus root	Cancer pain due to spleen deficiency, damp obstruction, and Qi stagnation	RCT	124 patients	Modified Xiangsha Liujunzi decoction enhanced the analgesic effect of transdermal fentanyl patches and reduced drug‐related adverse reactions.
Modified Shenling Baizhu decoction [65]	Codonopsis root, Astragalus root, Poria, Atractylodes rhizome (fried), common yam rhizome, white hyacinth bean seed, lotus seed, Coix seed, amomum fruit, platycodon root, medicated Leaven, Oldenlandia Diffusa, and licorice root	Cancer pain due to lung and spleen Qi deficiency with phlegm accumulation and toxin formation	RCT	60 patients	Modified Shenling Baizhu decoction significantly alleviated pain in patients with lung cancer bone metastasis.
Gutongling decoction [66, 67]	Drynaria rhizome, centipede, horny goat weed leaf, Pyrite, Aconite root (*A. carmichaelii*), and aconite root (*A. kusnezoffii*)	Kidney essence deficiency with blood stasis obstructing the meridians, which is associated with cancer pain.	RCT	100 patients	The combination of Gutongling Formula and Feiyanning decoction with Western medicine for the treatment of bone metastases in lung cancer significantly alleviated pain and improved the patient’s quality of life.

*Note*: Herbal medicines are presented using standardized English common names followed by their corresponding Latin binomial nomenclature, as follows: Coix seed (*Coix lacryma-jobi L.*); Angelica root (*Angelica sinensis (Oliv.) Diels*); Chuanxiong rhizome (*Ligusticum chuanxiong Hort.*); red Peony root (*Paeonia lactiflora Pall.*); Spatholobus stem (*Spatholobus suberectus Dunn*); curcuma root (*Curcuma wenyujin Y.H. Chen & C. Ling*); Pinellia rhizome (*Pinellia ternata (Thunb.) Breit.*); Inula flower (*Inula japonica Thunb.*); Oldenlandia Diffusa (*Hedyotis diffusa Willd.*); Actinidia root (*Actinidia chinensis Planch.*); Bupleurum root (*Bupleurum chinense DC.*); safflower (*Carthamus tinctorius L.*); bitter orange (*Citrus aurantium L.*); licorice root (*Glycyrrhiza uralensis Fisch.*); Achyranthes root (*Achyranthes bidentata Blume*); Platycodon root (*Platycodon grandiflorus (Jacq.) A. DC.*); Costus root (*Saussurea costus (Falc.) Lipsch.*); Amomum fruit (*Amomum villosum Lour.*); Atractylodes rhizome (*Atractylodes macrocephala Koidz.*); tangerine peel (*Citrus reticulata Blanco*); Cassia seed (*Cassia obtusifolia L.*); Common yam rhizome (*Dioscorea polystachya Turcz.*); white hyacinth bean Seed (*Lablab purpureus (L.) Sweet*); lotus seed (*Nelumbo nucifera Gaertn.*); medicated leaven (*Massa Medicata Fermentata, a fermented herbal preparation*); Drynaria rhizome (*Drynaria fortunei (J. Sm.) J. Sm.*); centipede (*Scolopendra subspinipes Latreille*); horny goat weed leaf (*Epimedium sagittatum (Sieb. & Zucc.) Maxim.*); pyrite (*Pyritum*).

Abbreviations: P‐RCT = prospective randomized controlled clinical trial, RCT = randomized controlled clinical trial.

#### 3.1.2. Critical Appraisal of Clinical Trials on Herbal Formulas for Cancer Pain

Critical appraisal of the clinical trials cited in Table 1 reveals inconsistent methodological rigor. While all studies were described as RCTs, reporting of key methodological details was often incomplete [68]. Randomization methods were frequently omitted, raising concerns about selection bias [69]. Sample sizes were generally modest (60–240 participants), potentially limiting statistical power for detecting significant differences in subjective outcomes such as pain relief [70]. Furthermore, blinding represents a challenge in herbal decoction trials, as creating a credible placebo control that matches taste and appearance is difficult [71–73]. None of the cited studies mentioned attempts at blinding participants or outcome assessors, increasing the risk of performance and detection bias. The choice of control groups was typically standard analgesic care, which is clinically relevant but does not control for the placebo effect inherent to TCM interventions [74]. Although sham acupuncture controls represent a methodological strength in trials of external therapies, this approach has not been extended to herbal medicine trials [75–78]. Future research should prioritize larger, multicenter trials with explicit reporting of randomization and allocation concealment methods, alongside innovative strategies for blinding where feasible.

#### 3.1.3. External TCM Therapies for the Management of Cancer Pain

Acupuncture alleviates pain by regulating Qi and blood flow and is widely used as an external TCM therapy for cancer pain [79, 80]. Acupuncture at the Siguan points (bilateral Taichong [LR3] and Hegu [LI4]), combined with Neiguan (PC6), Zusanli (ST36), and Sanyinjiao (SP6), regulates the Visceral Yin‐Yang and harmonizes the upper and lower body to effectively alleviate pain in advanced cancer patients [81]. For cancer pain attributed to kidney Yin deficiency with phlegm‐blood stasis, reinforcing and reducing techniques are employed: Taixi (KI3) and Xuanzhong (GB39) nourish kidney Yin, while additional points such as Fenglong (ST40), Xuehai (SP10), and SP6 resolve phlegm, activate blood, and eliminate stasis [82]. Clinically, combined moxibustion–acupuncture and moxibustion–auricular seed therapy are increasingly recommended for moderate to severe cancer pain [83–85].

Topical Chinese herbal medicine acts directly on pain sites to improve local Qi and blood circulation, making it an effective adjuvant therapy for localized cancer pain. Clinical evidence shows that combining conventional analgesics with topical applications improves pain intensity, frequency, duration, and quality of life indicators in patients with cancer pain [86]. Applying analgesic plasters containing Corydalis yanhusuo, Processed Nux Vomica (*Strychni semen praeparatum*), peach kernel (*Persicae semen*), safflower (*Carthami flos*), Sinomenium stem (*Caulis sinomenii*), and borneol (*Borneolum syntheticum*) to the Ashi point (localized pain), as well as performing acupuncture combined with topical plasters formulated with raw aconite (*Aconiti carmichaeli* Radix), Arisaema tuber (*Arisaematis rhizoma*), myrrh (*Myrrha*), frankincense (*Olibanum*), Spina Gleditsiae (*Gleditsiae Spina*), borneol, Shancigu (Pseudobulbus Cremastrae seu Pleiones) (*Cremastrae pseudobullbus*), and Gecko (*Gekko gecko*), can synergistically promote blood circulation, resolve stasis, unblock collaterals, and alleviate pain. These combined interventions effectively shorten the duration of cancer‐related pain and improve patients’ quality of life [87, 88].

Combined therapeutic approaches—such as topical Chinese herbal applications used with analgesics, moxibustion paired with auricular seed therapy, and acupuncture integrated with herbal medicine—have demonstrated notable analgesic effects in cancer pain management. In addition, other external TCM modalities, including herbal rubbing, herbal steaming, herbal enemas, nasal herbal drops, and herbal iontophoresis, also show potential benefits in alleviating cancer‐related pain [89–91].

#### 3.1.4. Convergent Biological Pathways in TCM Management of Cancer Pain

While TCM employs diverse herbs and therapeutic modalities for cancer pain management, emerging evidence reveals that many of these interventions converge on shared biological pathways. This synthesis provides a mechanistic framework for understanding how TCM achieves analgesic effects through multitarget approaches. Multiple TCM herbs and formulas target the arachidonic acid cascade, a key pathway in inflammatory pain. Compounds such as TSN IIA from Salvia miltiorrhiza, curcumin from Curcuma longa, and DHC from Corydalis yanhusuo demonstrate convergent inhibition of COX‐2 and PGE2 production [92, 93]. This shared mechanism underlies the TCM principle of “clearing heat and detoxification,” as these herbs collectively suppress NF‐κB signaling and reduce pro‐inflammatory cytokine release (TNF‐α, IL‐6, and IL‐1β) in both peripheral tissues and the central nervous system. Several analgesic herbs converge on TRP channel modulation. Corydalis alkaloids, aconitine derivatives, and curcumin all exhibit activity on TRPV1 and TRPA1 channels, which are critical mediators of thermal and mechanical hyperalgesia in cancer pain [94, 95]. Additionally, lappaconitine from Aconitum carmichaelii and TSN IIA modulate voltage‐gated sodium channels, reducing neuronal excitability and pain signal transmission [96]. These actions provide a molecular basis for the TCM concept of “unblocking collaterals.”

The TCM approach of “invigorating blood” finds mechanistic correlation with endogenous opioid system modulation. Compounds from Corydalis alkaloids, Curcuma, and Aconitum enhance the release of β‐endorphins and enkephalins while modulating μ‐opioid receptor sensitivity [97]. This convergent mechanism is particularly relevant for cancer‐induced bone pain, where these herbs restore opioidergic tone without the tolerance issues associated with exogenous opioids. Advanced cancer pain involves central sensitization through microglial activation. Multiple TCM interventions, including acupuncture and herbal compounds, converge on suppressing microglial P2X4 receptors and the subsequent brain‐derived neurotrophic factor (BDNF) release. Electroacupuncture at ST36 and LI4, combined with herbal components such as DHC, collectively inhibits p38 MAPK phosphorylation in spinal microglia, reducing central sensitization [98–101]. The TCM principle of “tonifying Qi” in pain management correlates with antioxidant mechanisms. Astragalus root compounds, ginsenosides, and TSNs converge on Nrf2/ARE pathway activation, enhancing antioxidant enzyme expression (SOD, CAT, and GPx) and reducing ROS accumulation in damaged neurons [102–104].

### 3.2. Cancer‐Related Fatigue and Chemotherapy‐Induced Fatigue

CRF is a common symptom in patients with advanced cancer, and it is classified under “Xu Lao” (deficiency syndrome) in TCM. The main symptoms of Xu Lao include physical weakness and emaciation, shortness of breath with a reluctance to speak, loss of appetite and indigestion, cold extremities, restlessness, and emotional instability. In TCM theory, Xu Lao is a weakened state caused by the excessive depletion of essence, Qi, blood, and body fluids. The treatment of debility primarily involves identifying the patient’s syndrome pattern to determine whether the therapeutic approach should address deficiency syndromes or mixed deficiency and excess conditions [105]. This shared TCM etiology allows for a unified therapeutic approach to fatigue, regardless of whether it originates primarily from the cancer or secondarily from its treatment.

#### 3.2.1. Herbal Medicine and TCM Formulas

The management of CRF, whether cancer‐related or chemotherapy‐induced, primarily employs strategies to strengthen the spleen and kidneys, tonify Qi and blood, and address both deficiency and excess conditions [105, 106]. Clinical studies have shown that TCM therapies, when used as adjuvant treatments, have potential advantages in managing chemotherapy‐induced adverse effects, including fatigue [107]. Tonifying herbs are the cornerstone of treatment. The most used herbs include Astragalus root, Ginseng root, and Codonopsis root [106, 108]. A clinical study at the University of Texas MD Anderson Cancer Center indicated that continuous oral administration of high‐dose Ginseng root (800 mg/day) for 29 days resulted in significant reductions in pain and fatigue scores [106]. Another study involving 60 patients with advanced‐stage CRF and spleen Qi deficiency syndrome demonstrated that a modified Gui Pi Tang (spleen‐restoring decoction), with Astragalus root as the principal herb, effectively alleviated clinical symptoms such as fatigue, reduced appetite, and loose stools [109]. Herbal formulas such as Jianpi Yishendecoction and Jianpi Shengsuiherbal paste have also been shown to significantly alleviate chemotherapy‐induced CRF [110, 111]. For cases presenting with mixed deficiency and excess, formulas such as Shuyu Pill, which simultaneously tonifies the body and dispels pathogenic factors, can be applied [86].

#### 3.2.2. External TCM Therapies

Acupuncture and related techniques are widely used to regulate spleen and kidney functions by stimulating relevant meridians, such as the Spleen Meridian of Foot‐Taiyin and the Conception Vessel. Commonly selected acupoints include ST36, SP6, KI3, and Qihai (CV6), with the aim of strengthening the spleen and stomach, enhancing kidney essence, and harmonizing the Zang‐Fu organs [112]. Studies have shown that acupuncture can improve the clinical features of CRF and reduce fatigue scale scores [112]. Notably, a randomized controlled trial demonstrated that true acupuncture at CV4, SP6, ST36, CV6, SP10, and PC6 acupoints was significantly more effective than sham acupuncture (inserting needles approximately 1 inch away from the actual acupoint) in alleviating postchemotherapy fatigue and improving immune function in patients with breast cancer [113].

Beyond acupuncture, other external modalities show promise. Stimulating the extraordinary points Sihuaxue (bilateral Geshu [BL17] and Danshu [BL19]) is effective in treating various deficiency diseases caused by Yin‐Yang imbalance and Qi–blood deficiency [114]. Combined approaches, such as Chinese herbal baths (with herbs such as Aconitum carmichaelii and cinnamon twig to tonify Qi and warm Yang) used together with electronic moxibustion at points such as ST36 and CV4, have been employed to warm Yang, tonify Qi, and promote the flow of meridians in advanced‐stage CRF [115]. Similarly, infrared laser moxibustion has been used to improve fatigue symptoms in breast cancer survivors [116].

#### 3.2.3. Critical Appraisal of Evidence for Fatigue Management

The evidence supporting TCM for fatigue management derives from studies targeting both cancer‐related and chemotherapy‐induced fatigue. A critical appraisal reveals a mixed methodological quality. A strength in several trials, such as the one cited above [113], is the use of sham acupuncture controls, which helps account for nonspecific effects and strengthens the validity of the findings specific to acupuncture. However, methodological limitations are common. The methods of randomization are often briefly described without details on allocation concealment, increasing the risk of selection bias. Sample sizes are generally small, with many trials enrolling fewer than 70 participants per group, potentially leading to underpowered studies. Blinding of participants and practitioners is challenging in trials involving procedures such as acupuncture, although blinding of outcome assessors is often feasible but was not consistently reported. Furthermore, the relatively short duration of some interventions may not be sufficient to evaluate long‐term efficacy. While the existing evidence is promising, these limitations necessitate that the findings be interpreted with caution. Future high‐quality RCTs with larger sample sizes, rigorous randomization and blinding procedures, and longer follow‐ups are warranted to establish robust evidence for TCM in managing cancer‐related and treatment‐induced fatigue.

### 3.3. GI Symptoms (Nausea, Vomiting, Anorexia, and Cachexia)

GI symptoms are among the most common and debilitating issues faced by patients with advanced cancer. Seventy to eighty percent of patients with advanced cancer experience symptoms, such as weight loss, reduced appetite, nausea, and vomiting, that are often associated with cancer anorexia–cachexia syndrome (CACS) [117, 118]. These symptoms not only stem from cancer but are also prominently induced by conventional treatments such as chemotherapy and radiotherapy, significantly affecting nutritional status, treatment tolerance, and overall quality of life. TCM approaches these symptoms holistically, aiming to regulate the spleen and stomach functions, harmonize the ascending and descending of Qi, and address the underlying patterns of deficiency and excess.

#### 3.3.1. Herbal Medicine and TCM Formulas

TCM employs herbal formulas to manage GI symptoms based on the specific syndrome presentation. For cancer‐induced anorexia and cachexia, which often manifest as spleen Qi deficiency, the therapeutic focus is on fortifying the spleen and harmonizing the stomach. Oral administration of a modified Sijunzi decoction, utilizing the method of tonifying Qi and harmonizing the stomach, has been shown to effectively improve anorexia and enhance the quality of life in patients with advanced gastric and colorectal cancer [119]. For chemotherapy‐induced nausea and vomiting (CINV), which can be associated with disharmony of the stomach Qi, formulas that regulate the middle energizer are used. A randomized study demonstrated that adding Liujunzi decoction to standard antiemetic therapy (paclitaxel + cisplatin) provided an additive effect in alleviating chemotherapy‐induced nausea, vomiting, and anorexia in patients with uterine cervical or corpus cancer [120, 121]. Similarly, the guideline from the Society for Integrative Oncology suggests evidence‐based support for integrative therapies for CINV [122, 123]. Other formulas, such as Xiangsha Liujunzi decoction, have also been reported to enhance the effect of transdermal fentanyl patches and reduce drug‐related adverse reactions, which may include GI upset [64].

#### 3.3.2. External TCM Therapies

External therapies offer noninvasive strategies for symptom control and are particularly valuable for patients with difficulty swallowing. For anorexia and dysregulation of Qi, acupoint application (herbal patching) at key points such as Shenque (CV8), Zhongwan (CV12), ST36, Pishu (BL20), PC6, and LR3 can help consolidate stomach Qi and regulate its flow, thereby improving appetite [119]. Acupuncture is a well‐established intervention. Acupuncture at PC6 is particularly renowned for managing CINV [124]. The 2017 guidelines from the Society for Integrative Oncology specifically recommend acupuncture to reduce CINV [125]. A common side effect of certain chemotherapies and opioids used for pain management, TCM offers topical solutions. Umbilical application of herbal medicines and the use of acupoint plasters combined with press needles have shown effectiveness in managing chemotherapy‐related constipation [126–128].

### 3.4. Sleep Disturbances (Insomnia)

Sleep disturbances, particularly insomnia, represent a prevalent and burdensome symptom cluster in patients with advanced cancer. These disturbances can arise from the psychological distress associated with the disease, uncontrolled physical symptoms such as pain, or a direct neurotoxic side effect of chemotherapy and other therapeutic agents. The presence of insomnia significantly compromises patients’ quality of life and cognitive function and may even influence treatment outcomes. Addressing sleep disturbances is therefore a critical component of comprehensive supportive care in oncology.

Among TCM modalities, acupuncture has emerged as a prominent nonpharmacological intervention for managing insomnia in cancer patients, including those specifically experiencing chemotherapy‐induced sleep disturbances [129]. The therapeutic mechanism is thought to involve the regulation of the autonomic nervous system and the modulation of neurotransmitters involved in the sleep–wake cycle. Evidence from a rigorously designed clinical trial supports its efficacy. An assessor–participant blinded, randomized, sham‐controlled trial investigated acupuncture for chemotherapy‐associated insomnia in breast cancer patients [129]. In this study, the intervention group received acupuncture at acupoints including Sishencong (EX‐HN1), Baihui (GV20), Shenting (GV24), PC6, KI3, and SP6, combined with auricular point pressing at the heart and Shenmen points. The control group received sham acupuncture along with pressing on three sham auricular points (Table 2).

**Table 2:** The application of traditional Chinese medicine to manage adverse reactions associated with chemotherapy and radiotherapy.

Adverse reaction type	Traditional Chinese Medicine intervention measures		Sample size		Trial plan	Treatment course	Research results
	Control group	Observation group	Control group	Observation group			
Fatigue [113]	Sham acupuncture (inserting needles approximately 1 inch away from the actual acupoint)	Acupuncture at CV4, SP6, ST36, CV6, SP10, and PC6 acupoints	68 patients	70 patients	Random assignment	14 days	Acupuncture treatment can effectively alleviate postchemotherapy fatigue and improve immune function in patients with breast cancer.
Gastrointestinal adverse reactions [120]	Paclitaxel + cisplatin + standard antiemetic therapy	Paclitaxel + cisplatin + standard antiemetic therapy + Liujunzi decoction	17 patients	19 patients	Random assignment	14 days	Liujunzi decoction alleviated chemotherapy‐induced nausea and vomiting.
Insomnia [129]	Sham acupuncture (needling at nonacupoints 1–2 cm near the actual acupoints) combined with pressing on three sham auricular points [123]	Acupuncture at EX‐HN1, GV20, GV24, PC6, KI3, and SP6 acupoints combined with auricular point pressing at the heart; Shenmen	69 patients	69 patients	Blinding method, randomization, and sham‐controlled trial method	126 days	Acupuncture effectively treated chemotherapy‐induced insomnia.
Myelosuppression [130]	Paclitaxel + cisplatin + antiemetic and antiallergic drugs	Paclitaxel + cisplatin + antiemetic and antiallergic drugs + moxibustion (bilateral ST36 and ST40 acupoints) + modified Bazhen decoction	60 patients	60 patients	Random number table method	73 days	Moxibustion combined with herbal medicine reduced the occurrence of myelosuppression in patients with solid malignant tumors undergoing chemotherapy.
Neutropenia [131]	Paclitaxel + carboplatin + rhG‐CSF	Paclitaxel + carboplatin + rhG‐CSF + Yi Qi ShengSui formula	30 patients	30 patients	Random number table method	21 days	The combination of the Yi Qi ShengSui formula and rhG‐CSF could potentially serve as an effective treatment option for chemotherapy‐induced neutropenia.
Toxic side effects [132]	Paclitaxel + cisplatin	Paclitaxel + cisplatin + modified Sha Shen Mai Dong decoction	32 patients	32 patients	Random chart method	42 days	The modified Sha Shen Mai Dong decoction combined with paclitaxel and cisplatin chemotherapy effectively reduced chemotherapy‐induced toxic side effects.

*Note:* Guanyuan (CV4); Sanyinjiao (SP6); Zusanli (ST36); Qihai (CV6); Xuehai (SP10); Neiguan (PC6); Dazhui (GV14); Yaoshu (GV2); Sishencong (EX‐HN1); Baihui (GV20); Shenting (GV24); Taixi (KI3); Fenglong (ST40).

Abbreviations: rhG‐CSF = recombinant human granulocyte stimulating factor injection.

In addition, moxibustion combined with herbal medicine has demonstrated therapeutic benefits in alleviating chemotherapy‐induced myelosuppression [130], while various Chinese herbal formulas have been shown to mitigate chemotherapy‐related toxicities—such as myelosuppression, GI dysfunction, and hepatic impairment—through multitarget mechanisms that regulate hematopoiesis, protect GI mucosa, and improve liver metabolism [131, 132]. Collectively, these findings suggest that integrated TCM approaches, combining acupuncture, moxibustion, and herbal medicine, may comprehensively address chemotherapy‐induced complications while improving sleep quality and overall treatment tolerance in cancer patients.

## 4. The Integration of TCM With Modern Medicine

The integration of TCM with Western medicine has emerged as an important trend in the management of advanced cancer. As outlined in Section 3, TCM contributes substantially to mitigating a wide spectrum of symptoms and treatment‐associated toxicities. Beyond its supportive role, growing evidence suggests that incorporating TCM into modern oncologic strategies—particularly immunotherapy—may enhance therapeutic efficacy while reducing adverse effects. This integrative approach further underscores the clinical relevance and expanding role of TCM in contemporary cancer care.

### 4.1. TCM Combined With Immunotherapy for the Treatment of Advanced Cancer

TCM can influence tumor immunity through various regulatory mechanisms [133]. Natural TCM products, such as curcumin, resveratrol, cardiac glycosides, polysaccharides, saponins, and capsaicin, as well as their derivatives, can act as immunomodulators in cancer treatment [133]. Studies have also shown that berberine can reduce the expression of programmed cell death 1 ligand 1 (PD‐L1) by inhibiting the deubiquitination activity of COP9 signalosome subunit 5 in nonsmall cell lung cancer, thereby promoting antitumor immunity [134]. Furthermore, the combination of the TCM formula Gegen Qinlian decoction with anti‐PD‐1 therapy can effectively restore T‐cell function by inhibiting checkpoint pathways [135]. Notably, the combination of TCM with immunotherapy (e.g., immune checkpoint inhibitors) not only enhances the efficacy of cancer immunotherapy but also reduces its side effects [136–139]. In parallel, accumulating mechanistic evidence demonstrates that a broad spectrum of TCM‐derived bioactive constituents—including flavonoids, saponins, alkaloids, and polysaccharides—exerts profound regulatory effects on the tumor microenvironment (TME). Given that the TME and tumor immunity engage in a bidirectional [140], dynamic interplay that drives immune evasion and therapeutic resistance, these compounds have been shown to reprogram tumor‐associated macrophages (TAMs) from a protumorigenic M2 phenotype toward an antitumor M1 state, attenuate the expansion of myeloid‐derived suppressor cells (MDSCs), normalize aberrant tumor vasculature, and promote cytotoxic CD8^+^ T‐cell infiltration [141]. Collectively, these microenvironmental modifications contribute to reversing TME‐mediated immunosuppression and enhancing the therapeutic responsiveness to immune checkpoint blockade.

In clinical practice, TCM injections as key components of modern Chinese medicine formulations, such as Elemene injection and Shenqi Fuzheng injection, have shown synergistic effects and reduced toxicity when combined with chemotherapy [142–144].

## 5. Future Perspectives and Conclusions

### 5.1. Current Limitations and Challenges

Although current findings suggest promising roles for TCM in the management of advanced cancer and cancer‐related symptom clusters, the evidence base remains limited by several methodological constraints. Many clinical studies suffer from small sample sizes, insufficient reporting of randomization procedures, inadequate allocation concealment, and suboptimal blinding—particularly in trials involving herbal decoctions [145]. These weaknesses introduce performance and detection bias and require cautious interpretation of results.

The intrinsic emphasis on individualized syndrome differentiation in TCM results in considerable heterogeneity in herbal formulations and acupuncture point prescriptions. While this reflects clinical reality, it complicates data synthesis and restricts the feasibility of robust meta‐analyses [146]. Mechanistic investigations are expanding, yet many continue to focus on isolated compounds or single signaling pathways. Such reductionist approaches are insufficient to delineate the complex synergistic interactions underlying multiherb formulas or the integrated neuro‐immune‐endocrine responses induced by acupuncture [147].

Moreover, the current literature lacks systematic pharmacovigilance and rigorous reporting of long‐term safety, particularly regarding herb–drug interactions with chemotherapy, targeted therapy, and immunotherapy. These gaps highlight the urgent need for methodological refinement and stronger mechanistic evidence to support clinical translation [148, 149].

### 5.2. Future Research Priorities

Patients with advanced cancer commonly experience pain, insomnia, and CRF, which are now increasingly recognized as active biological drivers of tumor progression rather than passive consequences of advanced disease. Persistent nociceptive input can facilitate tumor growth through sensory neuropeptide‐mediated pathways [144], and experimental sensory nerve ablation exerts both antinociceptive and antitumor effects [150]. Sensory neurons within the TME further modulate proliferation and metastasis [151], indicating a bidirectional neuro‐tumor interaction that may represent an underexplored therapeutic axis.

In parallel, sleep disturbances, circadian rhythm disruption, and sustained fatigue may accelerate tumor progression through systemic inflammatory signaling [152, 153]. Sensory neuropeptides—particularly substance P (SP) and calcitonin gene‐related peptide (CGRP)—play central roles in integrating nociceptive and immune pathways. CGRP induces CD8^+^ T‐cell exhaustion and M2‐type TAM polarization, promoting an immunosuppressive TME [154–157]. Although direct evidence linking SP to macrophage polarization is limited, its pro‐inflammatory properties suggest a potential role in shaping the TME. Together, these findings identify SP and CGRP as promising targets for dual‐action strategies aimed at alleviating nociception while mitigating immunosuppressive TME remodeling.

TCM‐derived bioactive constituents show potential to modulate these convergent neuro‐immune pathways. Compounds such as curcumin, berberine, resveratrol, saponins, and polysaccharides have been reported to regulate key signaling mechanisms, including NF‐κB, TRPV1, and the SP/CGRP–TAM axis [53, 54]. Prioritizing mechanistic validation of these constituents—particularly their potential synergy with immunotherapy—will be critical for advancing their translational relevance.

Nanotechnology offers an additional avenue for innovation. Nanocarriers incorporating curcumin, baicalin, xanthohumol, and related flavonoids exhibit improved pharmacokinetics, sustained release properties, enhanced TAM targeting, and reversal of chemoresistance [141, 158]. These platforms markedly increase the bioavailability of TCM compounds and provide a promising strategy for precision modulation of the TME and pain‐associated inflammatory networks.

To generate internationally robust evidence, future research should prioritize large‐scale, multicenter randomized controlled trials incorporating sham acupuncture arms, standardized placebo herbal preparations, validated patient‐reported outcomes, and transparent reporting in accordance with CONSORT‐CHM and STRICTA guidelines [159, 160]. Mechanistic studies integrating multiomics, network pharmacology, single‐cell transcriptomics, and advanced neuroimaging will be essential to elucidate the multicomponent‐multitarget mechanisms underpinning TCM interventions [161]. Moreover, pharmaco‐epidemiological investigations are required to characterize long‐term safety profiles and herb–drug interactions, particularly in combination with immunotherapy, thereby supporting the safe incorporation of TCM into contemporary oncology [149].

### 5.3. Final Conclusion

**Figure 1:**
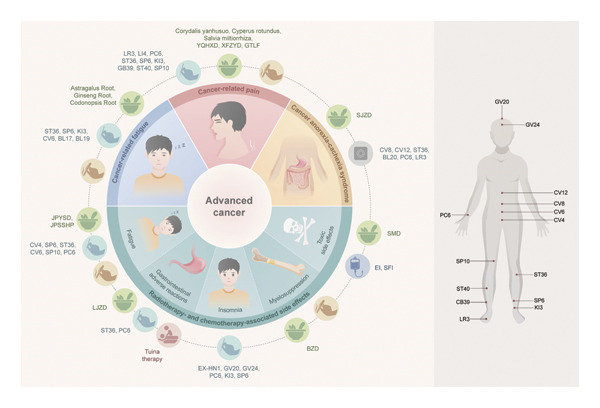
Overview of symptom alleviation strategies in advanced cancer with Traditional Chinese Medicine (TCM).

As illustrated in Figure 1, TCM provides individualized therapeutic frameworks tailored to patient constitution, disease stage, and pathological characteristics. Interventions such as herbal medicine, acupuncture, moxibustion, tuina, acupoint application, and combined acupuncture–herbal regimens are widely used to mitigate treatment‐related adverse effects—such as pain, fatigue, insomnia, GI dysfunction, myelosuppression, and neutropenia—and to enhance treatment tolerance and overall therapeutic outcomes. These interventions may also contribute to improving immune competence and maintaining functional status, ultimately enhancing quality of life for patients with advanced cancer.

Although substantial progress has been made, further high‐quality evidence is needed to confirm TCM’s effects on alleviating cancer‐related symptoms (cancer pain, fatigue, GI dysfunction, and chemotherapy/radiotherapy‐induced toxicities), providing supportive care and modulating immune function, define its mechanisms, and evaluate its long‐term safety profile. Continued basic and translational research will be essential for identifying novel therapeutic targets and advancing the integration of TCM into modern oncologic practice. A rigorous, mechanistically informed, and clinically grounded research agenda will enable TCM to play a more substantial and scientifically validated role in comprehensive cancer pain management.

TCM employs diverse therapeutic modalities, including traditional herbal decoctions, herbal injections, acupuncture, moxibustion, tuina, and acupoint therapy, to alleviate symptoms associated with advanced cancer. These approaches target cancer‐related pain, fatigue, GI discomfort, and adverse effects of chemotherapy and radiotherapy, offering holistic and integrative strategies for symptom management.

1. Common TCM herbs: Corydalis yanhusuo, Cyperus rotundus, Salvia miltiorrhiza, Astragalus root, Ginseng root, and Codonopsis root.
2. Common TCM formulas: YQHXD: Yiqi Huoxue decoction, XFZYD: Xuefu Zhuyu decoction, GTLF: Gutongling Formula, SJZD: modified Sijunzi decoction, JPYSD: Jianpi Yishen decoction; JPSSHP: Jianpi Shengsui herbal paste, LJZD: Liujunzi decoction, BZD: Bazhen decoction, and SMD: modified Shashen Maidong decoction.
3. Common TCM injections: Elemene injection (EI) and Shenqi Fuzheng injection (SFI).
4. Common acupuncture points: Taichong (LR3), Hegu (LI4), Neiguan (PC6), Zusanli (ST36), Sanyinjiao (SP6), Taixi (KI3), Xuanzhong (GB39), Fenglong (ST40), Xuehai (SP10), Qihai (CV6), Geshu (BL17), Danshu (BL19), Shenque (CV8), Zhongwan (CV12), Pishu (BL20), Guanyuan (CV4), Baihui (GV20), Shenting (GV24), and Sishencong (EX‐HN1).

## Ethics Statement

The authors have nothing to report.

## Consent

The authors have nothing to report.

## Disclosure

All authors read the manuscript and approved the final version of the manuscript.

## Conflicts of Interest

The authors declare no conflicts of interest.

## Author Contributions

Chunmeng Jiao: literature review, conceptualization, original draft writing, figure visualization, and contacted the Charlesworth Company to polish the manuscript. Ting Zhang: literature review and original draft writing, review, and editing. Chunmeng Jiao and Ting Zhang contributed equally to this work and share first authorship. Yanqing Wang: literature review, manuscript conceptualization, supervision, and manuscript editing. Chunmeng Jiao, Yachen Yang, Ruofan Zhang, Wenbo Liu, and Yanqing Wang: Manuscript revision.

## Funding

This work was supported by grants from the National Key Research and Development Program of China (2024YFC3505202) and the National Natural Science Foundation of China (82441055, 82474629, and 82271258).

## Data Availability

Data sharing is not applicable to this article as no datasets were generated or analyzed during the current study.
